# Effects of Culinary Spices on Liking and Consumption of Protein Rich Foods in Community-Dwelling Older Adults

**DOI:** 10.3390/nu15051172

**Published:** 2023-02-26

**Authors:** John C. Peters, Jeanne Anne Breen, Zhaoxing Pan

**Affiliations:** 1 Anschutz Health and Wellness Center, 12348 E. Montview Blvd., MailStop C263, Aurora, CO 80045, USA; 2 Division of Endocrinology, Diabetes and Metabolism, University of Colorado Denver, Anschutz Medical Campus, 12801 E. 17th Ave., RC1 South Rm 7103, Aurora, CO 80045, USA; 3 Department of Pediatrics, University of Colorado Denver, Anschutz Medical Campus, 13123 E. 16th Ave., B065, Aurora, CO 80045, USA

**Keywords:** older adults, meat and plant protein intake, herbs and spices, food liking, sensory acuity

## Abstract

Insufficient protein intake is a common challenge among older adults, leading to loss of muscle mass, decreased function and reduced quality of life. A protein intake of 0.4 g/kg body weight/meal is recommended to help prevent muscle loss. The purpose of this study was to assess whether the protein intake of 0.4 g/kg body weight/meal could be achieved with typical foods and whether culinary spices could enhance protein intake. A lunch meal test was conducted in 100 community-dwelling volunteers; 50 were served a meat entrée and 50 were served a vegetarian entrée with or without added culinary spices. Food consumption, liking and perceived flavor intensity were assessed using a randomized, two-period, within subjects crossover design. Within the meat or vegetarian treatments, there were no differences in entrée or meal intakes between spiced and non-spiced meals. Participants fed meat consumed 0.41 g protein/kg body weight/meal, while the vegetarian intake was 0.25 g protein/kg body weight/meal. The addition of spice to the vegetarian entrée significantly increased liking and flavor intensity of both the entrée and the entire meal, while spice addition only increased flavor for the meat offering. Culinary spices may be a useful tool to improve the liking and flavor of high-quality protein sources among older adults, especially when used with plant-based foods, although improving liking and flavor alone are insufficient to increase protein intake.

## 1. Introduction

Loss of muscle mass is an inevitable consequence of aging, with a 3–8% decline per decade after age 30 [1] and even greater losses after the age of 60 [2,3]. This can lead to frailty, osteoporosis and loss of physical function, diminishing the quality of life of older individuals [4,5]. Adequate nutrition, including sufficient energy and protein intakes as well as exercise, can help slow the rate of muscle decline [6,7]. Protein intakes that are insufficient to slow age-related muscle loss are a prevalent problem among older adults (≥60 years) [8]. Recent evidence also suggests that inadequate protein intake may contribute to loss of cognition with age [9].

Over the past decade, a number of experts and scientific advisory groups have evaluated the adequacy of protein intake recommendations for adults over the age of 60–65 [10,11,12,13,14]. In the US, for adults over the age of 60, the Recommended Dietary Allowance for protein intake established by the Food and Nutrition Board of the National Academy of Medicine is 0.8 g protein/kg body weight/d, which is the same as for younger adults [15]. The European Society for Clinical Nutrition and Metabolism (ESPEN) recommends 1–1.2 g protein/kg body weight/d for adults over the age of 65 to sustain muscle strength and function, and even greater amounts for individuals with severe illness or injury [13]. In 2017, the nutrition societies for Germany, Austria and Switzerland (D-A-CH) revised the reference intakes for protein for adults over the age of 65 to 1.0 g protein/kg body weight/d [12]. The PROT-AGE Study Group, a consortium of the European Union Geriatric Medicine Society (EUGMS), in cooperation with other scientific organizations, recommends an intake of 1–1.2 g protein/g body weight/d for adults over age 65 [10]. Additional insights developed in recent years have prompted independent experts to recommend a protein intake of at least 1.2 g protein/kg body weight for adults age >65 [11,16,17,18]. While there is a range of protein intake recommendations by authoritative sources from 0.8 to 1.2 g protein intake/kg body weight/d, the higher daily intake target is associated with more favorable conditions for maximal protein synthesis in older adults (e.g., [18]). The 1.2 g protein/kg body weight/d target equates to 0.4 g protein/kg body weight/meal, assuming a three-meal per day dietary pattern.

Inadequate protein intake among older adults may be caused by reduced appetite, a loss of sensory acuity with age, difficulty chewing tough and fibrous foods, the cost of high-quality protein sources, perceived intolerance to certain foods and other factors [19,20,21]. It may be possible to address the reduced appetite and sensory perception that occurs with age by enhancing the flavor intensity and liking of high-quality protein sources through the use of culinary herbs and spices [22,23]. In addition, preparing protein-rich foods that are easy to chew and swallow may facilitate greater consumption. Both improving food texture and increasing the flavor intensity and food liking of dietary protein sources while still adhering to dietary guidelines may contribute to increasing protein intake among older adults, helping improve physiological function and overall quality of life.

Few studies have specifically examined the role of culinary spices as a tool to improve protein intake among community-dwelling older adults. Published information from focus groups and quantitative surveys among community-dwelling older adults have suggested that increasing flavor would be advantageous to increasing intake [22,23]. This could be accomplished through addition of seasonings or adding sauces and gravies to protein entrees [24,25]. Other approaches could include adding flavor enhancers [26]. No studies to our knowledge have compared protein consumption from lunch meals with meat-based protein to those with plant-based protein sources. Likewise, we are not aware of studies in community-dwelling older adults that have assessed the potential effect of self-perceived declines in sensory acuity on protein intake and the effects of culinary spices on flavor perception, food liking and intake.

The purpose of the current study was to test whether a protein intake of 0.4 g/kg body weight could be achieved in a standardized meal and whether the addition of culinary spices could enhance protein intake compared to a meal without added spices. In addition, the study assessed whether the addition of culinary spices to a meal prepared with only salt and pepper improves liking and flavor intensity, which are often correlated with intake. The study looks at both a meat-based protein meal (M) and a plant-based protein (V) meal. This is important, as current dietary recommendations advocate consuming more plant-based diets [15], and this study can provide some perspective on the effectiveness of vegetable-derived protein sources for meeting the higher protein requirements of older individuals. Both of the test meal entrée recipes (M and V) were chosen to reduce the likelihood that food texture (e.g., fibrous, difficult to chew and swallow) would limit consumption. Information about liking, hunger and satiety also provide insight into how these factors may correlate with protein intake. Finally, we assessed flavor intensity as well as self-reported losses in smell and taste acuity to see if enhancing the flavor of high-protein dishes is associated with greater consumption in both individuals with and without reported sensory loss.

## 2. Materials and Methods

### 2.1. Design

A randomized, two-period, within subjects crossover design was used to examine and compare the consumption, liking, flavor intensity and subjective appetite ratings of two different lunch meal treatments in two separate participant groups fed different protein rich meals: 1. A meat-derived protein meal (M) and 2. A plant-derived (vegetarian) protein meal (V). The two test conditions for each protein meal type were (1) salt and pepper only preparation (No Spice, “NS”) as one meal and (2) herb and spice preparation with salt and pepper as the other meal (Spice, “S”). Within each protein meal type (M or V), the two test meals were matched as closely as possible for macronutrients and calories. A one-week washout period separated the two test meal treatments.

Sample size: There were limited data from intervention studies in the literature on which to base a power calculation. Previous studies of older adult populations have used treatment sample sizes of 18–50 individuals [22,25,27]. We used data from one large observational study that reported daily and meal-based protein intake to estimate sample size for this study [28]. Sample size was chosen to detect the efficacy of the S meal on protein consumption (g protein/kg body weight/meal), which was the primary outcome of interest. We estimated the standard deviation of protein intake (g/kg body weight/meal) to be 0.30 [28] and conservatively estimated the standard deviation of the individual difference between the two meal conditions as 1.414 × 0.30 = 0.424. We considered an increase by 0.2 g kg body weight^−1^ (i.e., 10 g more protein intake for a subject of 50 kg) for lunch protein intake to be of clinical importance. In this case, a sample size of 50 participants per protein source (100 total participants) ensures a 90% power at 5% significance to detect the intervention effect of meals enhanced with culinary spices. A sample size of 50 also provides a 90% power at 5% significance to detect a correlation of 0.44.

### 2.2. Participants

**Figure 1:**
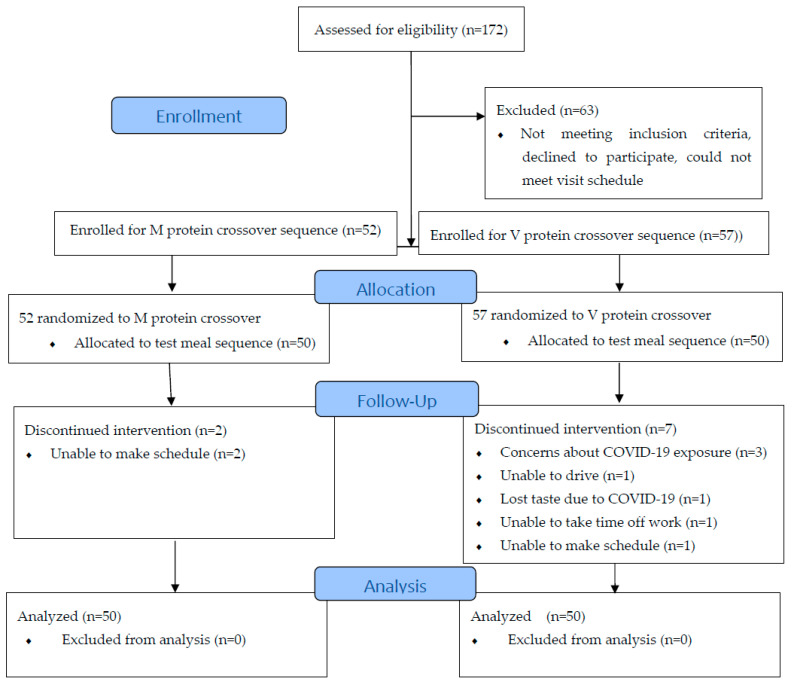
CONSORT Flow Diagram.

One hundred and nine community-dwelling male and female participants aged ≥60 years were enrolled in the study (52 for M treatment; 57 for V meal treatment), recruited from the Anschutz Medical Campus (drawing from over 17,000 employees on the campus) and Denver Metro area. Fifty participants in each treatment group completed the testing procedure (see CONSORT diagram in Figure 1). Participants were screened by telephone. Age 60 was selected as the lower age limit because this age is included in published literature on older adults when considering protein intake challenges (e.g., [8]). Participants of all body weights were accepted. Eligible participants had to be regular lunch consumers (≥5 days/week). Exclusion criteria were as follows: 1. diagnosed taste or sensory disorders that would prevent them from evaluating the food, 2. known eating disorders, 3. allergies to the test food/ingredients (including herbs and spices), 4. medications or medical conditions that may adversely affect taste (e.g., dysgeusia), 5. inability to complete the protocol, 6. personal dietary restrictions towards test meal items, and 7. dislike of the particular food items or herb/spices in the test meals. They were also asked their preference for being offered either the M or V meal entrée for testing (once the M group was filled, eligible participants were asked if they were okay with eating the V meal). Qualified individuals were assigned a schedule for their visits to the testing facility for participating in the study.

Participants in each treatment group (M or V) were randomized into a meal type sequence group (NS to S or S to NS), with a washout week in between each treatment.

All subjects provided informed consent for participation in the study. Participants received compensation having a value of USD 75 for completing both lunch visits.

### 2.3. Meal Testing

Testing consisted of serving complete meals at lunch and evaluating the consumption, liking and perceived flavor intensity of each item and the liking of the entire meal. In addition, we evaluated appetite before and after the test meals using a visual analog scale (VAS) instrument. Lunch was chosen as the eating occasion to study because it has been identified as an underutilized meal for leveraging protein intake [27].

For each test, the meals consisted of a protein entrée (M or V) and the same three accompanying side items (two butter lettuce leaves, two whole-wheat crackers and one half cup of seedless grapes). One version of the meal (NS) was prepared using only salt and/or pepper as added flavor elements (salt levels targeted to be consistent with current dietary recommendations) while the second version of the meal (S) was prepared with a more diverse blend of culinary spices to boost flavor. Test meals were served one week apart, aimed at determining the absolute liking of the meal/items vs. assessing relative liking by providing a side-by-side comparison at the same seating. This scheme is more relevant to a free-living condition in which people likely choose food items and recipes based on their absolute liking.

### 2.4. Test Meal Composition

The NS version of the M entrée consisted of chicken salad using commercially available canned, shredded skinless chicken breast, 2% fat yogurt, mayonnaise, celery, lemon juice and pepper. The S version of M entrée had the same composition as the NS version, but with added garlic powder, onion powder, ground mustard seed and dill. See recipes for both versions in Appendix A. Canned, skinless chicken breast was chosen as the protein source because it is a widely available, high-quality lean protein source that is familiar and well-liked by most people.

The NS version of the V entrée consisted of garbanzo beans (chickpeas) and brown rice, unflavored soy protein powder, 2% fat yogurt, mayonnaise, celery, lemon juice, water, salt and pepper. The S version had the same composition as the NS version plus added pepper, garlic powder, onion powder, ground mustard seed, parsley, oregano, dill, rosemary and cumin. See recipes for both V preparations in Appendix A. Addition of rice as a “complimentary protein” to the beans was necessary to ensure that the composite protein content of the entrée would have a complete indespensible amino acid profile. In addition, powdered soy protein was added (at a level that did not affect product acceptance and mouthfeel) to increase the protein density of the entrée.

We served entrée portions to each participant based on their body weight to facilitate consumption of at least 0.36 g/kg body weight/meal protein (representing 30% of a daily intake target of 1.2 g/kg body weight). We aimed to deliver between 18 and 40 g of protein in the meal entrée depending on body weight. We served an additional 20% of the entrée to ensure that intake was not constrained to the initial serving size. This would allow for consumption sufficient to meet the 0.4 g/kg body weight/meal level suggested for supporting optimal protein synthesis in older adults. For example, a 50 kg woman would be served an entrée containing 23.8 g protein, representing 55 kg × 0.36 g protein/kg body weight/meal × 1.2 (20% more) = 23.8 g protein/meal. If individuals consumed the entire entrée, they were free to ask for more. If requested, we provided additional portions (half the size of the first serving), and any remaining food was weighed back to determine energy and protein consumption.

### 2.5. Study Procedures

Participants were asked to eat their usual breakfast, but instructed to not eat any food or caloric beverages within 4 h of coming to the testing facility (The Anschutz Health and Wellness Center on the University of Colorado Denver, Anschutz Medical Campus in Aurora, Colorado). At their first visit, participants were weighed and their height was measured. Body weight information was used to calculate the entrée serving size as described earlier. Participants were assigned to individual tables, in a café environment with tables spaced six feet apart. Test meals were served at two times each test day: 11:30 a.m. and 1 p.m. Up to 20 participants were served on each test day, 10 at each seating. Participants were tested on the same day of the week and same time for the two test meals. Participants were blinded to the test conditions (meals with or without culinary spices), but because of the different visual appearance of the two entrees (the spiced version having green flecks), it was not possible to blind the study staff. Separate staff prepared and served the meals.

Once seated, participants were asked if there had been any change in their health status or other factors that may affect their ability to objectively complete the test meal. They were instructed on how to complete the meal liking and flavor intensity evaluations, VAS appetite measures [29] and VARSEEK [30] questionnaires. Participants were served the test meal for that visit according to the randomization treatment sequence schedule and were provided with 12 oz. of water to drink with the meal. Participants were allowed 45 min to complete the pre-meal assessments (VAS appetite assessment at both visits; sensory acuity and VARSEEK questionnaire at the first visit only), consume the meal and complete the post-meal assessments (VAS, liking and flavor intensity). All food items were weighed prior to serving and again after meal completion to assess protein and energy intakes.

Energy and protein intakes were calculated using the Nutrition Data System for Research database [31] based on the meal item composition (from the recipes) and the amounts consumed.

Assessments: On the first test day, prior to meal consumption, participants filled out a three-question assessment of self-perceived sensory acuity [32] (Appendix B 1). Participants also filled out a 9-point hedonic scale-rating instrument anchored by “dislike extremely” to “like extremely” that assessed their liking of the test entrée after the first few bites, liking after finishing the entrée and liking of the overall composite meal (Appendix B 2). Prior to meal presentation and after consumption of the meal, participants also completed a four-item, 100 mm visual analog scale (VAS) asking about their degree of hunger, satiety, fullness and prospective consumption feelings (Appendix B 3). The perceived flavor intensity of the entrée after the first few bites and after finishing it, and the rating for the entire meal was rated using a 5-point Likert Scale anchored by “no intensity at all” at one extreme and “extremely intense” at the other extreme (Appendix B 4). Finally, the VARSEEK questionnaire [30] was administered at the first seating prior to meal consumption as an exploratory probe to see if participants’ food-variety-seeking tendencies might correlate with consumption of the foods offered, given that the test foods may be perceived as not a part of their usual diet (Appendix B 5).

### 2.6. Data Analysis

Study data were double data entered into REDCap (Electronic Data Capture), a secure internal computerized database system [33]. The REDCap data comparison tool was used to compare and resolve discrepancies between the two copies of each subject’s records.

Participants’ demographic characteristics, self-reported sensory change since 35 years old and responses to the VARSEEK questionnaire were tabulated using summary statistics. All participants attended both sittings for each meal type so there were no missing data for the study.

Differences in protein and energy intakes, food liking, flavor intensity and satiety between test meal conditions (i.e., S or NS) were analyzed using a linear mixed effects model (LMM) with a random subject effect (Model 1). The LMM model consisted of the treatment sequence group (i.e., S->NS or NS->S), test meal period (i.e., visit 1 or visit 2), and test meal condition (i.e., S or NS) as fixed effects. Differences in the effect of spice addition between protein sources (M or V) was tested using a second model. In Model 2, we analyzed data from both entrée types together by introducing protein source (i.e., M or V protein) and its interaction term with test meal condition (i.e., S or NS) into Model 1 while allowing protein source to have different random subject effects. Under Model 2, the protein source effect was assessed by testing the interaction terms of protein source and meal condition. Separate exploratory analyses of any moderation effects of change in self-reported sensory acuity or food-variety-seeking tendency were conducted for the two protein sources. To investigate whether the spices’ effect was stronger in participants with vs. without decreased sensory acuity, we analyzed the data in the same fashion as Model 2 with sensory loss status substituting for protein source.

Other analyses: Individual scores for the difference between meal conditions in protein consumption were correlated with changes in liking scores, flavor intensity ratings, and hunger and satiety scores, respectively. In addition, the amount food consumed was correlated with the VARSEEK questionnaire composite score to assess any effect of food-variety-seeking behavior on intake. Finally, whether or not the participant had experienced COVID-19 (more than 6 months prior to testing) was correlated with protein intake. The appropriateness of normality assumptions was assessed by examining residual plots from the mixed model.

## 3. Results

### 3.1. Participant Characteristics

Characteristics of the 100 participants (50 for each meal test, all unique and not overlapping between treatments) enrolled in the study are shown in Table 1. There were no statistically significant differences between participants in the two meal treatment groups (M vs. V) in any of the characteristics measured, including age, sex, height, weight, body mass index (BMI), self-reported sensory acuity, COVID-19 history or responses to the VARSEEK questionnaire. It is noteworthy that both meal treatment groups had mean BMI values over 25, classifying them as having overweight or obesity. This is not surprising, as the prevalence of overweight and obesity among adults over the age of 20 in the USA is 73.9% [34].

### 3.2. Protein and Meal Consumption

Within each meal type, M or V, there were no significant differences in consumption of the entrée or total meal (when expressed as food weight or kcal) between the NS and S versions (Table 2). Likewise, there were no differences in the absolute amount of protein consumed or the amount eaten when expressed per kilogram of body weight for the NS or S versions of both the M and V meals. Energy (kcal) intakes of the entrée when expressed as a fraction of estimated total daily energy expenditure (TDEE, Table 1) were between 15 and 20% for both M and V meals. Values for the total lunch (including crackers and grapes) were between 21 and 26% of TDEE.

Participants served the M meal achieved total protein intakes of 32 ± 1.3 g and 32.7 ± 1.3 g/meal for NS and S versions, respectively (Table 2). Intakes expressed per kilogram of body weight were 0.40 ± 0.01 and 0.41 ± 0.01 g/meal for the NS and S versions, respectively. Seventy-four percent of M meal participants receiving NS achieved a protein intake ≥ 0.36 g/meal (30% of the daily target of 1.2 g/kg body weight/d for older adults), while 80% of those fed the S version ate at least 0.36 g/kg body weight/meal. These were not different between NS and S meals (*p* = 0.221). When considered against a target intake of 0.4 g/kg body weight/meal, 64% achieved the target for the NS entrée while 68% achieved the target for the S entrée (*p* = 0.534 for NS vs. S). Moreover, 93 and 94% of subjects served the NS and S meals, respectively, consumed at least 0.24 g/kg body weight/meal (*p* = 0.770 for S vs. NS), which equates to 30% of the current US RDA of 0.8 g/kg body weight/d (if lunch provided 30% of daily intake).

Protein intake from the V meal was 20 ± 1.4 g for both the NS and S versions (Table 2). Intakes per kilogram body weight were 0.25 ± 0.02 and 0.26 ± 0.02 g for the NS and S meals, respectively. Only 20 and 24% of participants receiving the NS and S V meals, respectively (*p* = 0.467), achieved a protein intake ≥0.36 g/kg body weight/meal. Compared to a target intake of 0.4 g/kg body weight/meal, 18% and 20% of participants achieved this level of intake for the NS and S meals, respectively (p=0.745). A much greater proportion of subjects, 48% and 60%, respectively, achieved a protein intake of 0.24 g/kg body weight/meal (*p* = 0.105), a level in line with the current US RDA.

There were no significant entrée by recipe (NS or S) interactions for the amounts/kcals of food consumed (Table 2).

### 3.3. Liking and Flavor Intensity

Liking scores for the M entrée and total meal ranged between 7.2 and 7.5 on a nine-point Likert scale for both the NS and S recipes (Table 3) and were not significantly different. This was true for the initial evaluation after a few bites of the entrée, for the post-meal evaluation of the entrée and for the entire meal. Liking scores for the V entrée and meal were somewhat lower for both NS and S versions compared to the M meal, ranging between 4.8 and 5.6 for NS and S versions, respectively (Table 3). Spice addition to the V entrée significantly increased liking at the beginning of the meal, at the end of the meal and for the overall meal (Table 3).

Flavor intensity ratings were significantly greater for the S version of both the M and V entrées compared to the NS recipes (Table 3) at both the beginning and end of the meal. Participants also rated the flavor intensity of the S version of the entire V meal significantly higher than that of the NS meal. There were significant entrée by recipe interactions for both liking and flavor intensity across all measures, meaning that the subjective responses to the addition of culinary spices were different between the M and V entrée types, with spices having a greater positive effect on V entrée and meal liking and flavor (Table 3).

### 3.4. Appetite Measures

There were no statistically significant differences in appetite measures before the meal between lunch types (M or V), nor were there differences in starting values between recipe types (NS or S) for each lunch type (data not shown).

The S version of the M meal was associated with a significantly greater reduction in prospective consumption following the meal compared to the NS meal (*p* = 0.047, Table 4). No other significant differences were observed for hunger, fullness or desire to eat between M and V meals for either NS or S versions of the entrees. There were no significant entrée by recipe interactions.

More than two-thirds of the participants rated their perception of food flavor and their sense of smell now compared to age 30–35 as being the same or stronger (Table 5), and two thirds rated their sense of smell now as being above average. Between 24 and 32% of participants rated these subjective variables as average or weaker than when they were younger.

Composite VARSEEK scores (range of 1–40, with higher scores indicating more likely to eat unfamiliar or novel items) were not different between the two meal treatment groups, with means of 28.09 ± 4.52 and 30.24 ± 4.72 for M and V groups, respectively (*p* = 0.176).

### 3.5. Moderator Effects

There were no significant effects of gender, BMI, sensory acuity, COVID-19 experience or VARSEEK score on the amount of M or V entrée consumed as a function of recipe type, NS or S. However, there were significant effects of gender, BMI, COVID-19 status, and sensory acuity on liking, flavor intensity and appetite scores.

Gender effects: For the M meal, perceived flavor intensity after a few bites was greater for the S version vs. the NS version among females (*p* = 0.031). In males, flavor intensity was greater for the S version of the M entrée after finishing the entrée and for the complete meal (*p* = 0.050 and *p* = 0.026, respectively).

BMI effects: Flavor intensity after a few bites was significantly greater among females with a BMI ≥25 (*p* < 0.005). Desire to eat and prospective consumption were lower for the S version of the M meal, respectively (*p* = 0.047 and *p* = 0.014), among participants with a BMI ≥25.

COVID-19 effects: Flavor intensity was greater for the S version of the M meal after a few bites (*p* = 0.008) and after finishing the meal (*p* = 0.039) among participants reporting never having COVID-19 vs. those who had it more than 6 months prior to the study.

Sensory acuity: For participants served the M meal, perceived flavor intensity was greater after a few bites and after finishing the meal (*p* = 0.028 and *p* = 0.012, respectively) among those reporting the same or stronger flavor perception now than when they were 30–35 years old. Likewise, reported flavor intensity was greater among those served the S version of the M meal, who rated their sense of smell now as above average or greater than when they were 30–35 years old.

For the V meal, there were a number of significant effects of self-assessed sensory acuity measures on liking and perceived flavor intensity (Table 6).

Participants with above average or better than average perceived smell rated liking and flavor of the S version of the V entrée and meal significantly greater than the NS version. Those with average or below average perceived smell liked the S version significantly more than the NS version for initial liking and flavor intensity and for final flavor intensity. Ratings of liking and flavor intensity were significantly higher for S compared to NS versions of the entrée and meal when participants rated smell the same or stronger now compared to when they were 30–35 years old. Those with perceived weaker smell also rated initial flavor intensity greater for the S version, but other liking and flavor scores were not different between NS and S versions. Perceived flavor sensitivity effects showed a pattern similar to that of smell, with participants reporting the same or stronger flavor sensitivity having significantly greater liking and flavor intensity scores for the S version of the entrée and meal compared to the NS version. Initial entrée flavor intensity and final flavor intensity of the entrée and meal were significantly greater among participants who rated their flavor sensitivity weaker than when they were younger.

## 4. Discussion

A major objective of this study was to investigate whether protein intake at a single meal could meet or exceed the recommended per meal protein intake for community-dwelling older persons to maintain muscle mass and function. An additional aim was to determine whether the addition of culinary spices could improve liking of high-quality protein foods, potentially increasing their consumption to help meet this protein intake target. A daily protein intake of 1.2–2 g protein/kg body weight/d is recommended to preserve muscle mass and prevent loss of function in older adults [13,18,36,37,38]. This equates to a per meal intake of 25–35 g of high-quality protein, assuming a three meal per day consumption pattern [13,39,40]. Furthermore, when expressed on a body weight basis, an intake of 0.4 g protein/kg body weight/meal or greater is suggested to trigger protein synthesis [41].

In this study, we observed protein intakes for the M meal of > 30 g/meal that met the 25–30 g total protein intake per meal and the 0.4 g/kg body weight/meal targets for roughly two-thirds of participants. In contrast, participants served the V meal consumed a mean of only 20 g of protein, and only one in five met the 0.4 g/kg body weight/meal target. These findings are not surprising given that the protein density of the M meal was nearly three times greater (0.167 g protein/g M entrée vs. 0.062 g protein/g V entrée). Even with the addition of soy protein powder to the V entrée, it was not possible to match the greater protein density of the M entrée. Participants served the V entrée would have had to consume substantially greater volumes in order to achieve the protein intake target, although the portions served were large enough to accomplish this. In the present study, participants fed the V meal actually ate 25% more energy than those fed the M meal, but it was still insufficient to meet the protein intake target. Both treatment groups ate to the same level of satiety (Table 4). The greater protein density and complete indespensible amino acid profile of meat-based vs. vegetable-based sources [42,43,44] is the main reason that it was difficult to match protein and amino acid densities between M and V foods. Furthermore, in an attempt to make the foods easy to chew and swallow, we chose the chicken and bean/rice salad forms, whose recipes had other ingredients that diluted the protein content.

Surprisingly, the addition of culinary spices to either the M or V entrée did not increase consumption beyond that observed with addition of salt and pepper alone. Spice addition significantly improved liking scores for the V entrée, and meal and flavor intensity scores were greater for both M and V entrees and for the overall V meal. Food liking is often correlated with consumption [45,46], although this is not a consistent finding [47,48,49]. Adding seasonings to meals of older adults has been shown to increase flavor intensity perception and intakes of protein, energy and fat [24,25]. This can occur even in the absence of changes in perceived pleasantness [24], providing evidence that increasing liking is not a pre-requisite for increased intake.

In the case of the M entrée, it is possible that the base recipe, with the flavors of chicken, mayonnaise, yogurt, celery and pepper, was sufficiently pleasant to a make further increase in liking less probable. Absolute liking scores for the NS version of the M entrée averaged 7.2–7.4, which is typical for foods that are well liked [50], and these relatively high liking scores leave little room for an increase upon the addition of spices. For the V entrée, absolute liking scores were significantly lower than for the M entrée, averaging 4.8–5.3 for the NS version, and the addition of spices significantly improved both liking and perceived flavor intensity. There was a significant interaction between the effect of spice addition to either the M or V meals, with spices increasing liking for the V meal more than for the M meal. This may not be surprising, as the V meal composed of beans and rice was less flavorful than the M meal, which had the savory flavor of chicken as the foundation.

Furthermore, 24 to 32% of our participants reported having weaker smell and flavor sensitivity than when they were 30–35 years old, while 32% reported average or below average smell acuity at present. These numbers are consistent with other studies of age-related changes in sensory acuity. Rawal and colleagues [32] found that 16% of older women reported below average smell function while the overall occurrence of smell and flavor sensory dysfunction was 30%. Similarly, in other surveys, the incidence of self-reported taste and smell alterations among adults over age 40 was 23% for smell and 19% for taste [51]. In the present study, participants fed the M meal and who reported the same or stronger flavor perception and above average smell than when they were younger scored the S version of the entrée and meal as more intense, while those reporting weaker flavor perception did not. This suggests that the participants with reduced subjective sensory acuity were less able to detect the intensity of the added spices. Even greater differences were found for the V meal (Table 6) showing consistently greater liking and flavor intensity among those with no reported decline in sensory acuity and weaker effects among those reporting sensory decline. This suggests that adding spices to the relatively bland V entrée created a more discernable contrast to the NS version compared to adding spices to the more savory M entrée.

Declining sensory acuity has been suggested to be one factor affecting the loss of appetite, reduced food intake and reduced food liking among older adults [19,21,52,53]. Focus groups and quantitative surveys among older adults suggested that boosting liking and “tastiness” would help promote protein intake [23,54], and adding seasonings is one strategy for accomplishing this [25,55]. Furthermore, food familiarity and other cognitive cues may be helpful in maintaining food intake in the face of declining sensory acuity [56,57]. Our findings support this previous work and indicate that adding spices to high quality protein foods can improve flavor, and sometimes liking, but other factors may drive total food and protein intakes.

Previous COVID-19 status (having the virus more than 6 months prior to study) did not affect the consumption of either the M or V meals. Although sensory losses from COVID-19 are reportedly restored within a few months [58], this may not be true for everyone, with 1 in 10 affected people reporting little improvement after 2 years [59]. In our study, flavor intensity was reportedly greater for the S version of the M meal among those who never reported having COVID-19. It is possible that those who previously had COVID-19 still suffered from some sensory impairment, although not severe enough for them to report it upon screening.

Variety-seeking behavior among individuals is one factor that may affect the selection and intake of foods [30], and the VARSEEK instrument was developed to assess this tendency. We used the VARSEEK instrument to investigate whether variety seeking behavior was associated with the consumption of either NS or S versions of the M and V entrées. The scores of 29–31 obtained for our sample are reflective of medium variety seekers, with scores of below 25 or above 35 indicating either low or high variety seeking, respectively. There was no interaction between these scores and intake suggesting that the foods we served were neither unfamiliar nor particularly novel, which might have affected intake.

This study is encouraging in that it shows it is possible to achieve the proposed protein intake target of 0.4 g protein/kg body weight/meal for older individuals, at least for meat-based entrees. This was accomplished with ingredients that are readily available, affordable (canned chicken breast, etc.) and easily prepared at home. It also points to the challenges of achieving the same high level of protein intake when plant-based protein sources are used. While achieving adequate protein intakes from plant-based diets is possible for younger individuals whose total food and energy intakes are higher than for older adults, the lower protein per kcal density of plant proteins makes it much more difficult for older adults whose protein requirements are greater [44,60,61]. Because most plant proteins do not contain all indispensable amino acids, it is necessary to mix protein sources (e.g., beans and rice) which thus reduces the overall protein density of the food. Even when the V entrée was fortified by adding soy protein powder, it was hard to increase the protein density enough to ensure intakes that would meet the 0.4 g/kg body weight/meal target without negatively affecting mouthfeel. Soy protein powder could be used only in limited amounts due to its astringency and graininess.

Despite the lack of effect of culinary spices on increasing total protein intake in the present study, these substances have bioactive properties beyond their ability to enhance the flavor of foods. Culinary spices such as oregano, rosemary, cumin, parsley and dill used in the present study are rich in polyphenolic compounds [62,63,64] having significant antimicrobial, antioxidant, anti-inflammatory, anti-diabetic and anticancer properties [65]. Thus, the use of culinary spices to boost flavor and improve liking of foods may deliver benefits to adults of all ages.

Study strengths and limitations: Strengths of this study include a relatively large sample size for a randomized and standardized meal test, objective measures of intake and subjective measures of liking, flavor intensity, appetite and subjective sensory acuity. Test foods were selected that were familiar and could easily be prepared at home from readily available and affordable ingredients. The test food formulations also eliminated potential problems related to chewing and swallowing, which are issues more common among older individuals. In effect, the food forms served were optimized for promoting easy consumption, providing a reasonable benchmark for testing the feasibility of achieving the higher protein intake target for older individuals. A significant limitation of this study is that it tested only one meal occasion, lunch, and further studies would be needed to determine whether total daily protein intake could be sustainably increased to meet the 1.2 g protein/kg body weight/day target for older individuals. We did not collect information about the dietary intake of participants either before or after the test lunch, so we could not estimate total daily protein intake. Finally, the particular choice of foods and spices tested is a limitation given the much wider variety of foods consumed as part of a normal diet.

## 5. Conclusions

This study demonstrates that it is feasible to achieve protein intakes of ≥30 g/meal and 0.4 g/kg body weight/meal in community-dwelling older adults using high-quality meat-based foods that are easy to chew and swallow. Achieving this high level of protein intake from plant sources is more difficult and a selection of foods very high in protein density or fortified with complete protein would be required. Culinary spices can improve flavor intensity and liking of protein-rich foods, but do not necessarily improve protein intake. Further studies are necessary to explore the potential of culinary spices to support greater protein intake among older adults.

## Figures and Tables

**Table 1:** Participant Characteristics.

Characteristic	Chicken Lunch (*n* = 50)	Bean and Rice Lunch (*n* = 50)
Sex, *n (%)*	Female 39 (78) Male 11 (12)	Female 36 (72) Male 14 (28)
Age, mean *± SD* ^1^ (range) Age, median	68.7 ± 6.3 (60–81) 68	70.7 ± 7.4 (60–87) 70.5
Ethnicity *n, (%)*	Hispanic/Latino 5 (10) Non-Hisp./Latino 44 (88) Other 2 (4)	Hispanic/Latino 6 (12) Non-Hisp./Latino 41 (82) Other 3 (6)
Race *n, (%)*	White 48 (96) Other 2 (4)	White 42 (84) African American 2 (4) Asian 1 (2) Native American 1 (2) Other 4 (8)
Body Weight (kg), mean *± SD*	80.4 ± 13.6	79.1 ± 16.0
^2^ BMI (kg/m^2^), mean *± SD*	30.2 ± 5.1	28.9 ± 5.1
^3^ TDEE for light activity level	1864.4 ± 274.0	1866.5 ± 339.6
^4^ Sense of smell now, mean *± SD*	5.64 ± 1.44	5.58 ± 1.3
^5^ Sense of smell compared to when 30–35 years old, mean ± *SD*	3.84 ± 0.91	3.78 ± 0.71
^5^ Sense of flavor now compared to when 30–35 years old, mean ± *SD*	4.00 ± 0.70	4.00 ± 0.83
Has subject ever had COVID-19? (*n*, %)	No 37 (74.0%)	No 34 (68%)
	Yes 13 (26.0%)	Yes 16 (32%)
If history of COVID-19, did COVID-19 affect subject’s taste, smell or appetite in the last 6 months? (*n*, %)	No 13 (100%)	No 15 (94%)
		Yes 1 (6%)
If history of COVID-19 affected taste orsmell previously (but not in last 6 months), are effects ongoing? (*n*, %)	No 6 (100%)	No 4 (100%)
If history of COVID-19 affected appetite (but not in last 6 months), are appetite effects ongoing? (*n*, %)	No 3 (100%)	No 4 (100%)
^6^ Total VARSEEK score, mean ± *SD*	29.98 ± 4.52	30.24 ± 4.72

^1^ *SD*, Standard deviation; ^2^ BMI, Body mass index; ^3^ TDEE, Total Daily Energy Expenditure (as estimated by the Miflin, St Jeor equation, assuming light activity [35]); ^4^ Score on a 7-point Likert Scale, anchored by “Very Poor” and “Excellent”; ^5^ Scores on a 7-point Likert Scale, anchored by “Extremely Weaker” to “Extremely Stronger”. ^6^ The larger the VARSEEK score (range 1–40), the more likely to eat unfamiliar or novel items.

**Table 2:** Food and protein consumption.

Outcome	Type of Entrée Served	LS Mean (SEM), No Spice	LS Mean (SEM), Spice	*p* for within Entrée Comparison, NS vs. S	*p* for Entrée by Recipe Interaction
Amount entrée eaten (g)	Chicken salad	181.40 (7.54)	185.36 (7.54)	0.510	0.340
	Bean/rice salad	296.38 (21.57)	282.38 (21.57)	0.432	
Water consumed (g)	Chicken Salad	240.10 (16.18)	240.76 (16.27)	0.957	0.268
	Bean/rice Salad	230.96 (14.41)	252.14 (14.41)	0.133	
Kcal entrée eaten	Chicken Salad	278.15 (11.56)	284.22 (11.56)	0.510	0.733
	Bean/rice Salad	369.27 (27.37)	367.09 (27.37)	0.923	
Kcal entrée eaten as % of TDEE (from Table 1)	Chicken Salad	14.87 (0.52)	15.26 (0.52)	0.410	0.983
	Bean/rice Salad	19.66 (1.43)	20.08 (1.43)	0.732	
Grams protein consumed from entrée	Chicken Salad	30.24 (1.26)	30.90 (1.26)	0.510	0.661
	Bean/rice Salad	18.24 (1.36)	18.25 (1.36)	0.995	
Grams protein consumed, total meal	Chicken Salad	31.97 (1.29)	32.66 (1.29)	0.489	0.655
	Bean/rice Salad	19.97 (1.38)	19.99 (1.38)	0.982	
Total protein consumed, g/kg body weight	Chicken Salad	0.40 (0.01)	0.41 (0.01)	0.526	0.997
	Bean/rice Salad	0.25 (0.02)	0.26 (0.02)	0.585	

**Table 3:** ^1^ Liking and Flavor Intensity Scores.

Outcome Measure	Type of Entrée Served	LS Mean (SEM), No Spice	LS Mean (SEM), Spice	^2^*p* for within Entrée Comparison, NS vs. S	*p* for Entrée by Recipe Interaction
Liking Rating of entree (after eating a few bites)	Chicken Salad	7.40 (0.18)	7.46 (0.18)	0.760	**<0.001**
	Bean/rice Salad	5.28 (0.27)	6.56 (0.27)	**<0.001**	
Flavor intensity of entree (after eating a few bites)	Chicken Salad	2.26 (0.12)	2.60 (0.12)	**0.016**	**0.016**
	Bean/rice Salad	1.56 (0.13)	2.38 (0.13)	**<0.001**	
Liking Rating of entree (after finishing lunch meal)	Chicken Salad	7.16 (0.20)	7.36 (0.20)	0.407	**0.004**
	Bean/rice Salad	4.78 (0.29)	6.04 (0.29)	**<0.001**	
Liking Rating of overall meal (after finishing lunch meal)	Chicken Salad	7.22 (0.20)	7.36 (0.20)	0.533	**0.009**
	Bean/rice Salad	5.26 (0.26)	6.28 (0.26)	**<0.001**	
Flavor intensity of entree (after finishing lunch meal)	Chicken Salad	2.26 (0.13)	2.56 (0.13)	**0.047**	**0.017**
	Bean/rice Salad	1.54 (0.14)	2.36 (0.14)	**<0.001**	
Flavor intensity of overall meal (after finishing lunch meal)	Chicken Salad	2.44 (0.12)	2.48 (0.12)	0.766	**0.003**
	Bean/rice Salad	1.74 (0.13)	2.38 (0.13)	**<0.001**	

^1^ Liking scores are from a 9-point Likert scale anchored by “dislike extremely” to “like extremely”. Flavor intensity scores are from a 5-point Likert scale anchored by “no intensity at all” to “extremely intense”. ^2^ Values in bold are statistically significant *p* < 0.05.

**Table 4:** Appetite measures.

Visual Analog Question (Scored in mm from 0 to 100 mm)	Type of Entree Served	LS Mean (mm ± SEM), No Spice	LS Mean (mm ± SEM), Spice	^1^*p* for within Entrée Comparison, NS vs. S	*p* for Entrée by Recipe Interaction
How hungry now rating (after finishing lunch meal)	Chicken Salad	14.56 (2.40)	11.73 (2.40)	0.176	0.223
	Bean/rice Salad	14.14 (2.94)	15.58 (2.92)	0.610	
How full now rating (after finishing lunch meal)	Chicken Salad	65.58 (3.48)	71.16 (3.48)	0.145	0.745
	Bean/rice Salad	69.33 (3.76)	73.16 (3.72)	0.323	
How strong desire to eat rating (after finishing lunch meal)	Chicken Salad	15.84 (2.51)	12.09 (2.51)	0.161	0.204
	Bean/rice Salad	13.22 (3.03)	14.95 (3.00)	0.610	
How much could eat rating (after finishing lunch meal)	Chicken Salad	17.86 (2.38)	12.57 (2.38)	**0.047**	0.449
	Bean/rice Salad	15.03 (2.54)	12.64 (2.52)	0.395	

^1^ Values in bold are statistically significant *p* < 0.05.

**Table 5:** Subjective Sensory Acuity.

Sensory Acuity Measure	Category	Count (Percent)	95% Lower CI	95% Upper CI
Rating the flavor of food now compared to when you were 30 to 35 years old?	Same/stronger	38 (76.0%)	61.8%	86.9%
	Weaker	12 (24.0%)	13.1%	38.2%
Rating of sense of smell now	Above average/higher	34 (68.0%)	53.3%	80.5%
	Average/below	16 (32.0%)	19.5%	46.7%
Rating of sense of smell compared to when 30 to 35 years old	Same/stronger	34 (68.0%)	53.3%	80.5%
	Weaker	16 (32.0%)	19.5%	46.7%

**Table 6:** V meal; *p* values for significant differences between NS and S versions on liking and perceived flavor intensity with sensory acuity within a subgroup. There was no significant interaction (moderation) effect except for flavor of food vs. liking entrée rating. Participants gave higher ratings for S version than for NS for each comparison.

	Perceived Sensory Acuity Rating	Initial Entrée Liking	Initial Flavor Intensity	Final Entrée Liking	Overall Meal Liking	Final Entree Flavor Intensity	Overall Meal Flavor Intensity
Rating of sense of smell now	Above average/higher	<0.001	<0.001	<0.001	<0.001	<0.001	<0.001
	Average/below	0.007	0.010			0.028	
Sense of smell compared to when 30 to 35 years old	Same/stronger	<0.001	<0.001	<0.001	<0.001	<0.001	<0.001
	Weaker		0.030				
Flavor of food compared to when 30 to 35 years old	Same/stronger	<0.001	<0.001	<0.001	<0.001	<0.001	0.002
	Weaker		0.005			0.012	<0.001

## Data Availability

Data described in this article can be requested by any qualified researcher and will be provided upon reasonable request.

## Appendix A

M and V Entrée Recipes, NS and S Versions

**Chicken Salad Entree**			
***No Spice Version (NS)***		***Spiced Version (S)***	
INGREDIENTS	Wt. (g)	INGREDIENTS	Wt. (g)
Hellmann’s Mayonnaise	80	Hellmann’s Mayonnaise	80
Fage Greek Yogurt, 2% fat	80	Fage Greek Yogurt, 2% fat	80
Lemon Juice, fresh	6.3	Lemon Juice, fresh	6.3
Black Pepper	0.43	Black Pepper	0.8
Kirkland Canned Chicken, 270 mg sodium/serving	454	Garlic Powder	6
Celery, diced	70	Onion Powder	8
		Mustard Seed, ground	3.5
		Dill	1.2
		Kirkland Canned Chicken, 270 mg sodium/serving	454
		Celery, diced	70
**Chickpea and Rice Salad**			
***No Spice Version (NS)***		***Spiced Version (S)***	
INGREDIENTS	Wt (g)	INGREDIENTS	Wt (g)
Hellman’s Mayonnaise	80	Hellman’s Mayonnaise	80
Fage Greek Yogurt, 2% fat	80	Fage Greek Yogurt, 2% fat	80
Lemon Juice, fresh	12.8	Lemon Juice, fresh	13
Water	100	Water	100
Morton Iodized Salt	1	Morton Iodized Salt	1
Black Pepper	0.43	Black Pepper	2
365 Everyday Garbanzo Beans	240	365 Everyday Garbanzo Beans	240
Brown Rice	168	Brown Rice	168
Bob’s Red Mill Soy Protein Powder	28	Bob’s Red Mill Soy Protein Powder	28
Celery, diced	70	Celery, diced	70
		Garlic Powder	6
		Onion Powder	6
		Parsley	0.6
		Oregano	0.5
		Dill	0.6
		Cumin	0.7
		Mustard seed, ground	0.7
		Rosemary, ground	0.2

## Appendix B

Questionnaires and Visual Analog Appetite Measures

B-1: Self-rated olfactory function

How would you rate your sense of smell right now?

Very Poor	Poor	Below Average	Average	Above Average	Good	Excellent
❑	❑	❑	❑	❑	❑	❑

How would you rate your sense of smell now compared to when you were 30 to 35 years old?

Extremely Weaker	Much Weaker	Somewhat Weaker	The Same	Somewhat Stronger	Much Stronger	Extremely Stronger
❑	❑	❑	❑	❑	❑	❑

How would you rate your ability to sense the flavor of food now compared to when you were 30 to 35 years old?

Extremely Weaker	Much Weaker	Somewhat Weaker	The Same	Somewhat Stronger	Much Stronger	Extremely Stronger
❑	❑	❑	❑	❑	❑	❑

B-2: Food Liking

Thank you for participating in this study. We would like to get your opinion about the meal you have just eaten. Please answer the following questions to best of your ability.

How much do you like or dislike the entrée meal item?

Dislike extremely	Dislike very much	Dislike moderately	Dislike slightly	Neither like nor dislike	Like slightly	Like moderately	Like very much	Like extremely
❑	❑	❑	❑	❑	❑	❑	❑	❑

How much do you like or dislike this overall meal?

Dislike extremely	Dislike very much	Dislike moderately	Dislike slightly	Neither like nor dislike	Like slightly	Like moderately	Like very much	Like extremely
❑	❑	❑	❑	❑	❑	❑	❑	❑

B-3: Flavor Intensity

Please rate the flavor intensity of the entrée meal item.

No Intensity at all	Slightly Intense	Moderately Intense	Very Intense	Extremely Intense
❑	❑	❑	❑	❑

Please rate the flavor intensity of the overall meal.

No Intensity at all	Slightly Intense	Moderately Intense	Very Intense	Extremely Intense
❑	❑	❑	❑	❑

B-4: Visual Analog Appetite Rating Scales (VAS)

B-5: VARSEEK Questionnaire

1. When I eat out, I like to try the most unusual items, even if I am not sure I would like them.

Strongly Disagree	Disagree	Neither Disagree or Agree	Agree	Strongly Agree
❑	❑	❑	❑	❑

2. When preparing foods or snacks, I like to try out new recipes.

Strongly Disagree	Disagree	Neither Disagree or Agree	Agree	Strongly Agree
❑	❑	❑	❑	❑

3. I think it is fun to try out food items one is not familiar with.

Strongly Disagree	Disagree	Neither Disagree or Agree	Agree	Strongly Agree
❑	❑	❑	❑	❑

4. I am eager to know what kind of foods people from other countries eat.

Strongly Disagree	Disagree	Neither Disagree or Agree	Agree	Strongly Agree
❑	❑	❑	❑	❑

5. I like to eat exotic foods.

Strongly Disagree	Disagree	Neither Disagree or Agree	Agree	Strongly Agree
❑	❑	❑	❑	❑

6. Items on the menu that I am unfamiliar with make me curious.

Strongly Disagree	Disagree	Neither Disagree or Agree	Agree	Strongly Agree
❑	❑	❑	❑	❑

7. I prefer to eat food products I am used to.

Strongly Disagree	Disagree	Neither Disagree or Agree	Agree	Strongly Agree
❑	❑	❑	❑	❑

8. I am curious about food products I am not familiar with.

Strongly Disagree	Disagree	Neither Disagree or Agree	Agree	Strongly Agree
❑	❑	❑	❑	❑
